# Plier Ligands for Trapping Neurotransmitters into Complexes for Sensitive Analysis by SERS Spectroscopy

**DOI:** 10.3390/bios13010124

**Published:** 2023-01-11

**Authors:** Olga E. Eremina, Olesya O. Kapitanova, Alexei V. Medved'ko, Alexandra S. Zelenetskaya, Bayirta V. Egorova, Tatyana N. Shekhovtsova, Sergey Z. Vatsadze, Irina A. Veselova

**Affiliations:** 1 Chemistry Department, Moscow State University, Moscow 119991, Russia; 2 N.D. Zelinsky Institute of Organic Chemistry, Russian Academy of Sciences, Moscow 119991, Russia

**Keywords:** catecholamines, dopamine, neurotransmitter, resonance Raman spectroscopy, silver nanoparticles, surface-enhanced Raman spectroscopy (SERS)

## Abstract

Catecholamines–dopamine, noradrenaline and adrenaline are important biomarkers of neurotransmitter metabolism, indicating neuroendocrine tumors and neurodegenerative diseases. Surface-enhanced Raman spectroscopy (SERS) is a promising analytical technique with unprecedented multiplexing capabilities. However, not all important analytes exhibit strong SERS signals on stable and robust nanostructured substrates. In this work, we propose a novel indicator system based on the formation of mixed ligand complexes with bispidine-based bis-azole ligands which can serve as pliers to trap Cu(II) ions and stabilize its complexes with catecholamines. Four synthesized ligands with different functional groups: carboxyl, amino, benzyl, and methoxybenzyl, were applied for forming stable complexes to shift maximum absorbance of catecholamines from the ultraviolet region to 570–600 nm. A new absorbance band in the visible range resonates with the local surface plasmon resonance (LSPR) band of metal nanoparticles and most used laser wavelengths. This match allowed use of Molecular Immobilization and Resonant Raman Amplification by Complex-Loaded Enhancers (MIRRACLE) methodology to measure intense Raman signals on a nanostructured silver-based SERS-active substrate. The synthesized plier-like ligands fixed and stabilized catecholamine complexes with Cu(II) on the SERS sensor surface, which facilitated the determination of dopamine in a 3.2 × 10^−12^–1 × 10^−8^ M concentration range.

## 1. Introduction

Neurotransmitter metabolism (or metabolism of biogenic amines) forms the basis of neural mediation both in the peripheral and central nervous systems. The concentration of biogenic amines varies in normal and pathological conditions, which allows them to be used as molecular diagnostic markers both in basic research and in clinical medical practice. Catecholamines (CAs), such as dopamine (DA), noradrenaline (NA), adrenaline (AD), their precursor–l-dioxyphenylalanine (L-DOPA), and metabolites–homovanillic (HVA) and vanillylmandelic acids (VMA) are biologically active substances involved in the regulation of nervous activity, the cardiovascular system, the lipid peroxidation system, energy metabolism and myocardial contractility, in microcirculation and tissue oxygen supply, and in the regulation of embryogenesis [1]. NA in blood plasma serves as a prognostic biomarker for disorders in the functioning of the cardiovascular system [2]. The ratios of CAs and their metabolites in the urine are used to diagnose an adrenal tumor–pheochromocytoma [3]. Deviations in the synthesis and metabolism of CAs lie in the etiology of depression and related conditions [1]. However, there are two main groups of diseases associated with impaired neurotransmitter metabolism. The first group includes neurodegenerative disorders characterized by the death of neuronal cells in various areas of the brain and spinal cord, which corresponds to a decrease in the content of CA and their metabolites in the body. The main types of such diseases include Alzheimer’s (AD), Parkinson’s (PD), Huntington’s disease and amyotrophic lateral sclerosis [4]. The second group of diseases includes neuroendocrine disorders–neuroblastoma, pheochromocytoma, paraganglioma, carcinoid tumors, which are caused by excessive CA secretion and irritation of the autonomic nervous system [5]. Biochemical low-molecular markers of various diseases are associated with disorders of neurotransmitter metabolism in living organisms in clinical diagnostics are the precursor (L-DOPA), catecholamines (DA, AD, and NA) and their final metabolites (HMA and HVA).

The first major challenge for detecting and quantifying CAs in blood plasma or other biological fluids is that they are normally present at very low concentrations (at the level of 1 nM and lower). Moreover, with various pathological disorders, such as AD and PD, the concentration of CAs in biological samples decreases by an order of magnitude or more. The second important aspect of reliable diagnosis is the possibility of simultaneous selective determination of several analytes in a single sample [1]. Notably, in the blood, CAs can be quickly oxidized by platelet monoamine oxidases; thus, the determination of CAs in the body should be ideally carried out rather quickly, within 15–30 min [6]. Several analytical methods have been described to measure CAs and related biogenic amines in biological fluids, e.g., blood, urine, and cerebrospinal fluid. The most common methods are electrochemical [7,8,9,10], enzyme immunoassay [11,12,13], fluorometric [6,14,15], high-performance liquid chromatography (HPLC) [16,17,18] and capillary electrophoresis (CE) [19,20,21]. Even though these methods have good selectivity and low limits of detection (LODs), they cannot always be used for the multiplex determination of CA. Furthermore, simultaneous quantification of catecholamines and related biogenic amines remains an analytical challenge because of their low concentrations in plasma, the oxidation-prone catechol moiety, potential chromatographic interferences, and poor fragmentation characteristics in the mass spectrometer [22,23]. De facto, most of these methods are laborious, time-consuming, relatively imprecise, require bulk instrumentation and sample pretreatment steps with oxidation-sensitive CAs, and use large sample volumes [22]. Chemical sensors and biosensors have been among the most demanded tools of modern analytical chemistry over the past decades compared to relatively bulky, expensive and complex analytical instruments [24]. Currently, the most widely used in clinical diagnostics are electrochemical sensors, due to their high efficiency, portability, simplicity, and relatively low cost. However, as a rule, electrochemical sensors do not allow simultaneous, performance, i.e., multiplexed detection of several target analytes in one sample.

Surface-enhanced Raman spectroscopy (SERS) is widely used in the fields of chemical and biological analysis as a fast, sensitive, highly selective, and informative technique for the qualitative and semiquantitative determination of amino acids, nucleotides, water-soluble and membrane proteins, and nucleic acids [25,26,27,28,29,30]. In one of the first works on SERS determination of CAs, the multiplex determination of DA and NA was carried out in model solutions with non-modified silver nanoparticles (AgNPs) [31]. Despite the similarity of the SERS spectra of DA and NA, clear differences in the SERS spectra in the region of 1271–1325 cm^−1^, associated with the adsorption behavior of CAs on the surface of AgNPs, were reported. The achieved LODs were 5 nM for DA and NA, and addition of 0.5 wt% of albumin into the analyzed sample had no effect on the SERS spectra of CA. Moody and Sharma demonstrated multiplexed determinations of seven neurotransmitters: melatonin, serotonin, glutamate, DA, GABA, NA, and AD, by SERS with gold and silver nanoparticles in combination using 532, 633, and 785 nm laser wavelengths [32]. The reported LODs were 0.2, 1, and 10 μM for DA, NA, and AD, respectively. To increase the sensitivity of CA determination and match required for diagnosis LODS, one can vary the size and shape of nanoparticles, as well as modify chemical design of nanoparticle surface. For instance, ultrasensitive SERS detection of DA and serotonin down to 0.1 nM was demonstrated on graphene-gold heterostructural nanopyramids [33]. The additional 10-fold increase in the SERS intensity supposedly occurred as a result of chemical amplification originating from π-π interactions between graphene and aromatic ring structures in DA and serotonin. Thus, SERS is a promising technique for the multiplexed sensitive determination of CAs and related biogenic amines, but few quantitative methods have been proposed so far, most of which are based on the determination of only one compound. Therefore, the development and search for new sensing approaches to the determination of neurotransmitters is one of important tasks for chemical analysis.

Herein, we propose a novel indicator system based on plier-like ligands for trapping catecholamines into stable, colored complexes with Cu(II) ions. We synthesized four plier-like bispidine-based bis-azole ligands with different substituents: carboxylic, amino, benzyl, and methoxybenzyl groups. The stability of the obtained complexes with Cu(II) was studied with spectrophotometry and potentiometry. Importantly, the formation of ternary complexes allowed measurement of strong SERS signals on the silver nanostructured substrate. High sensitivity was achieved as a result of applying Molecular Immobilization and Resonant Raman Amplification by Complex-Loaded Enhancers (MIRRACLE) methodology, as the ligands and Cu(II) allowed to shift the absorbance of catecholamines from the ultraviolet into visible range which provided additional signal gain.

## 2. Materials and Methods

### 2.1. Materials

Dopamine hydrochloride (≥98%), norepinephrine (or noradrenaline, ≥98%), epinephrine (or adrenaline, ≥98%), chitosan (CS, with Mw∼150 kDa and a degree of deacetylation of 85%), and copper sulfate (CuSO_4_·5H_2_O were purchased from Sigma-Aldrich, USA. Hydrochloric, nitric, and acetic acids were obtained from Khimmed, Russia. ACS grade solvents were purchased from Khimmed, Russia, and used as received. All aqueous solutions were prepared using deionized water with a specific resistance of at least 18.2 MΩ·cm (Millipore, Martillac, France).

### 2.2. Synthesis of Bispidine-Based Bis-Azole Ligands

General procedure for [3+2]-cycloaddition. To a solution of 1,5-dimethyl-3,7-di(prop-2-yn-1-yl)-3,7-diazabicyclo[3.3.1]nonan-9-one (1 mmol) and respective azide (2 mmol) in 10 mL of tert-butanol the solutions of sodium ascorbate (0.2 mmol) in 2.5 mL of water and CuSO_4_·5H_2_O (0.1 mmol) in 2.5 mL of water were added sequentially. The reaction mixture was stirred for 36 h at room temperature under argon. The solution was evaporated to dryness and quenched with 30 mL of dichloromethane (DCM) and 10 mL of water. The organic layer was separated and washed with water until discoloration of water layer. The organic phase was dried with sodium sulfate and evaporated to dryness.

tBu2L^1^. White foam. Yield 80%. ^1^H-NMR (400 MHz, CDCl_3_, δ, ppm) 0.93 (s, 6H, CH_3_); 1.48 (s, 18H), 2.42 (d, 4H, 10.8 Hz), 3.05 (d, 4H, 10.8 Hz), 3.69 (s, 4H), 5.08 (s, 4H), 7.66 (s, 2H). ^13^C-NMR (100 MHz, CDCl_3_, δ, ppm) 19.68, 27.91, 46.57, 51.43, 51.83, 65.10, 83.55, 124.24, 145.09, 165.37, 215.21. HRMS-ESI. Calculated for [C_27_H_42_N_8_O_5_+H^+^]: 559.3351. Found: 559.3356

Boc2L^2^. White foam. Yield 56%. Purified with gradient column chromatography eluting from DCM:MeOH 10:1 to DCM:MeOH 3:1 and to DCM:MeOH:25% NH_3_·H_2_O 1:1:0.01. ^1^H-NMR (400 MHz, DMSO-d_6_, δ, ppm) 0.82 (s, 6H, CH_3_), 1.30 (s, 18H, Boc), 2.32 (d, 4H, 10.5 Hz), 3.00 (d, 4H, 10.6 Hz), 3.33–3.36 (t, 4H, 5.7 Hz), 3.59–3.64 (m, overlapped with H_2_O), 4.36 (t, 4H, 5.7 Hz), 7.86 (s, 2H). ^13^C-NMR (100 MHz, DMSO-d_6_, δ, ppm) 20.12, 28.44, 40.37, 46.31, 49.48, 51.38, 64.70, 78.46, 124.20, 143.52, 155.92, 217.88. HRMS-ESI. Calculated for [C_31_H_38_N_8_O_8_+H^+^]: 571.3145. Found: 571.3145.

L^3^. Yield 83%. All data are in accordance with our previous work [34].

L^4^. White powder. Yield 71%. ^1^H-NMR (400 MHz, CDCl_3_, δ, ppm) 0.89 (s, 6H, CH_3_); 2.37 (d, 4H, 10.9 Hz), 2.98 (d, 4H, 10.9 Hz), 3.62 (s, 4H), 3.79 (s, 6H), 5.46 (s, 4H), 6.88 (d, 4H, 8.9 Hz), 7.23 (d, 4H, 8.9 Hz), 7.44 (s, 2H). ^13^C-NMR (100 MHz, CDCl_3_, δ, ppm) 19.68, 46.56, 51.96, 53.59, 55.29, 65.14, 114.15, 114.37, 122.45, 126.83, 129.53, 129.71, 145.23, 159.81. HRMS-ESI. Calculated for [C_29_H_48_N_10_O_5_+H^+^]: 617.3887. Found: 617.3882.

General procedure for Boc-deprotection. Cooled TFA (56.0 mmol) was added to cooled Boc-protected compound (0.56 mmol) in dry DCM (5 mL) and the resulting solution was stirred overnight at room temperature. The solution was evaporated to dryness, the residue was dissolved in 5 mL of DCM and evaporated to dryness again. The dissolving-evaporation procedure was repeated 3 times. The resulting oil was triturated with dry diethyl ether till formation of powder. The latter was filtered, washed with dry diethyl ether, and dried in vacuo over P_2_O_5_.

L1·2TFA. White hygroscopic powder. Yield 97%. ^1^H-NMR (400 MHz, D_2_O, δ, ppm, mixture of ketone and gem-diol) 0.77, 0.82 (s, 6H, CH_3_); 2.85–2.97 (m, 6.5H), 3.46 (d, 1.5H, 11.2 Hz), 3.92 (s, 2.5H), 4.03 (s, 1.5 H), 5.24 (s, 4H), 8.03, 8.05 (s, 2H). ^13^C-NMR (D_2_O, mixture of ketone and gem-diol) 13.83, 14.52, 39.23, 45.40, 48.96, 49.32, 50.44, 59.00, 62.60, 92.99, 111.34, 114.24, 117.13, 119.67 (JC-F 291 Hz), 127.07, 127.12, 139.01, 139.23, 161.87, 162.22 (JC-F 35 Hz), 169.62, 169.69, 210.86. Calculated for C_19_H_26_N_8_O_5_·2C_2_F_3_O_2_H: C 40.96, H 4.18, N 16.61. Found: C 41.26 H 4.61 N 16.55.

L2·3.3TFA. White hygroscopic powder. Yield 95%. ^1^H-NMR (400 MHz, D_2_O, δ, ppm, mixture of ketone and gem-diol) 0.82, 0.89 (s, 6H, CH_3_), 2.88–3.05 (m, 6.85H), 3.51–3.60 (m, 5.50H), 4.02 (s, 2.75H), 4.16 (s, 1.25H), 8.11, 8.13 (s, 2H). ^13^C-NMR (D_2_O, mixture of ketone and gem-diol) 13.59, 13.87, 38.37, 39.20, 45.47, 46.90, 48.84, 49.07, 58.81, 62.42, 93.04, 111.49, 114.37, 117.27, 120.17 (JC-F 292 Hz), 126.30, 126.38, 138.90, 139.17, 162.22, 162.57 (JC-F 35 Hz), 210.72. Calculated for C_19_H_32_N_10_O·3.3C_2_F_3_O_2_H: C 38.78, H 4.49, N 17.67. Found: C 38.86 H 4.92 N 17.66.

### 2.3. SERS Sensor Fabrication

A SERS-active AgNP-based planar substrate was prepared by a universal approach as previously reported [35,36]. Briefly, to 100 mL of freshly prepared 0.17 mM aqueous silver nitrate solution (Carl-Roth, Karlsruhe, Germany) 30 mL of 2.5 M sodium hydroxide solution (Sigma-Aldrich, St. Louis, MO, USA) was added dropwise for complete precipitation of a dark brown silver(I) oxide. The prepared oxide then was washed 5 times with water and completely dissolved in 35 mL of 30% aqueous ammonia solution (Sigma-Aldrich, USA). To obtain a 0.1 M solution of [Ag(NH_3_)_2_]OH we added water to 170 mL and then diluted it to prepare final 12.5 mM solution of silver(I) complex. In the ultrasonic silver rain deposition process, this initial ammonia solution of [Ag(NH_3_)_2_]OH was nebulized into mist. AgNPs were deposited on a preheated surface of a glass slide (290–320 °C) in the form of droplets of 1–5 mm in size, which then evaporated, forming “coffee rings”. Silver nanoparticles formed aggregates with average size 185 ± 6 nm (see Supplementary Information, Figure S12). In addition, to immobilize the target analytes–catecholamines, for their preconcentration and uniform distribution, the silver surface was additionally modified with an optically transparent polymer film–chitosan (CS) 1.7 ± 0.1 μm thick. This feature of chitosan film was demonstrated by our group previously [36,37,38].

### 2.4. Immobilization of Cu(II) Ions and Bispidine-Based Bis-Azole Ligands into Chitosan Film

The chitosan film was prepared by drop-casting 10 μL of a 1 wt% CS solution in 1vol% acetic acid. After drying at room temperature for at least 2 h, 10 μL of a mixture of Cu(II) sulfate with one of the studied bispidine-based bis-azole ligands: L^1^, L^2^, L^3^ or L^4^, was applied onto a CS-coated AgNPs surface. Then, the prepared modified sensor was left drying at room temperature for at least 1 h.

### 2.5. Material Characterization and Raman Measurements

The obtained substrates were examined by XRD measurements using a Rigaku D/MAX 2500 machine (Tokyo, Japan) with a rotating copper anode (Cu Kα irradiation, 5–90° 2θ range, 0.02° step). Diffraction maxima were indexed using the PDF-2 database. UV−vis absorption spectra were recorded using the Lambda 950 (PerkinElmer, Waltham, MA, USA) UV−vis spectrophotometer with an attached diffuse reflectance accessory. Measurements were performed in the spectral range of 250–850 nm with a scan step of 1 nm. The obtained nanostructured substrates were characterized by scanning electron microscopy (SEM) Carl Zeiss NVision 40 and transmission electron microscopy (TEM) combined with electron diffraction (ED) on a 215 LEO 912 AB OMEGA, Carl Zeiss setup at 100 kV accelerating voltage. To perform Raman measurements, an InVia Raman confocal microscope (Renishaw, Wotton-under-Edge, UK) was used. All SERS spectra were acquired using a 17 mW 633 nm argon laser with a power neutral density filter of 10%. The spectra were collected using a 50× objective lens and with 10 s of acquisition time. A silicon wafer was used for instrument calibration.

## 3. Results and Discussion

### 3.1. Complexes of Catecholamines with Cu(II) Ions

Catecholamines are known to form complexes with transition metal ions [39,40,41]. Some of these complexes have a band with a high molar absorption coefficient in the 500–750 nm region, which is characteristic of the intramolecular charge transfer. The formation of complexes that absorb light in the visible region allows additional resonance enhancement in SERS [42]. First, we studied the possibility of the formation of complexes of dopamine, norepinephrine, or adrenaline with metal ions: Mg(II), Al(III), Cu(II), Co(II), Ni(II), Fe(II), and Fe (III) in a concentration ratio of 1:1 (see Supplementary Information file, Figure S1). The obtained UV-vis spectra of aqueous solutions of CAs with the metal ions showed that high molar absorption coefficients in the region of 750–900 nm are characteristic of CA complexes with Cu(II) ions. Moreover, compared to Fe(III), whose complexes showed similar molar absorption coefficients in this region, Cu(II) ions in aqueous solutions do not have such pronounced oxidizing ability and react only with strong reducing agents, which as a rule are absent in the biological fluids. In addition, copper-containing reagents are widely available, and the resulting complex compounds are quite stable.

**Figure 1:**
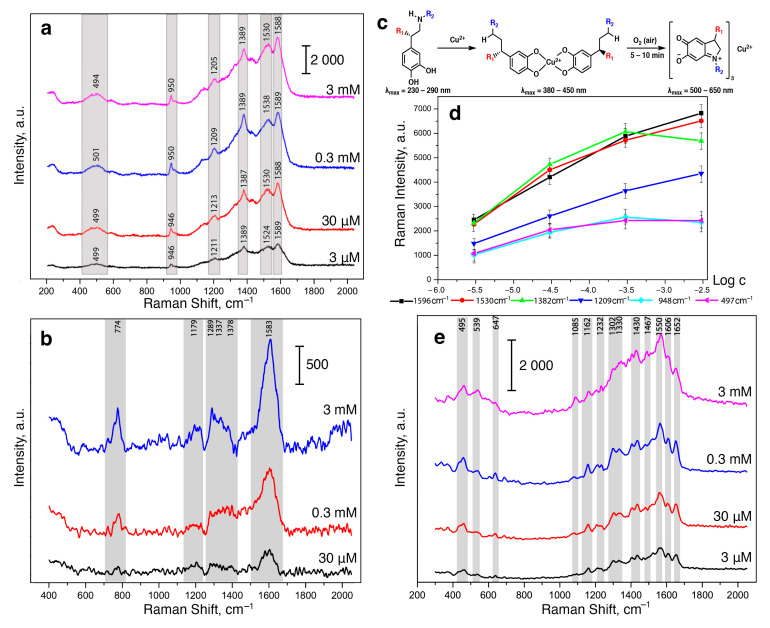
(**a**) SERS spectra of DA at 3 mM, 0.3 mM, 30 μM, and 3 μM concentrations with 3 mM Cu(II). (**b**) SERS spectra of NA at 3 mM, 0.3 mM, and 30 μM concentrations with 3 mM Cu(II). (**c**) Scheme of (Cu(semiquinone)_3_)^−^ formation [45]. (**d**) Dependence of the intensity of SERS signals (at 1596, 1530, 1382, 1209, 948 and 497 cm^−1^) on the logarithm of the DA concentration (M) in the presence of Cu^2+^ (3 mM). Error bars are shown for *n* = 20, *p* = 0.95. (**e**) SERS spectra of AD at 3 mM, 0.3 mM, 30 μM, and 3 μM concentrations with 3 mM Cu(II). Spectra were measured on the silver nanostructured surface coated with the chitosan layer at 633 nm laser wavelength.

First, we studied the possibility of measuring SERS spectra of catecholamine complexes. A previously developed and characterized SERS-active platform consisted of AgNPs adhered onto a glass slide and coated with a chitosan layer [36]. However, the application of a DA solution onto the SERS substrate did not result in high-intensity characteristic signals in SERS spectra [43]. This could be explained by the mismatch of the maximum absorption of CAs in UV region (230–300 nm) and the local surface plasmon resonance (LSPR) position of the obtained stable AgNP-based substrate. Thus, for the “visualization” of CA characteristic bands, we proposed to use catecholamine complexes that are capable of intensively absorbing in the visible spectrum (450–800 nm). We called this approach as Molecular Immobilization and Resonant Raman Amplification by Complex-Loaded Enhancers (MIRRACLE) [43]. Therefore, the SERS sensor surface was modified with a Cu(II) salt solution, then a model DA, AD or NA solution was applied onto the sensor and the Cu(II)–CA complex was formed in the CS layer (Figure 1). The SERS spectra for DA, AD, and NA in the form of their respective complexes with Cu(II) demonstrated intense characteristic bands: 1596 cm^−1^ (ν (C–C)_ring_), 1530 cm^−1^ (ν (C–C)), 1382 cm^−1^ (ν (C1–C2) + ν (C=O)), 1209 cm^−1^ (ν (C–H) + ν (C=O)), 948 cm^−1^ (ν (C–C) + ν (C=O)), and 497 cm^−1^ (ν (Cu_ring_). The obtained SERS spectra had vibrations characteristic of the (Cu(semiquinone)_3_)^–^ complexes formed on the AgNP surface. The peaks shown in Figure 1 agree with theoretically calculated and previously published bands [44], representing a “molecular fingerprint”. Furthermore, we observed lower signal enhancement and signal-to-noise ratio for AD and NA compared to DA.

When the concentration of CAs in the solution applied to the SERS-sensor element was changed, the peak positions did not shift but the intensities of each of the observed signals changed. However, due to the instability of the resulting complexes and the oxidation of CAs on the AgNP surface, the dependences of the intensities of the observed signals could not be linearized even in logarithmic coordinates (Figure 1d). This could be connected to the formation of CA complexes with copper ions of various stoichiometry. Moreover, UV-vis spectra of the CA complexes with Cu(II) demonstrated a gradual increase in optical density at a ca. 450 nm and a decrease at ca. 750 nm during the formation of complexes of the (Cu(semiquinone)_3_)^−^ type (see Supplementary Information File, Figure S2). The described change can be explained by the oxidation of catecholamines to the corresponding catecholates (Figure 1c). It should be noted that the oxidation of DA, NA, and AD with atmospheric oxygen, additionally catalyzed by copper ions, was completed within 15 min after mixing the solutions at mM concentrations.

The formation of such semiquinone Cu(II) complexes, on the one hand, is of interest since it is accompanied by a bathochromic shift of the absorption maxima for CAs. Thus, the resonance SERS effect can be achieved because of the overlap of the absorption maximum of the CA and the LSPR band of AgNPs and laser wavelength, which allow measurement of intense SERS signals. However, on the other hand, for future applications, when analyzing a real biological sample of a biological fluid, the selectively of CA determination in the presence of their metabolites (formed in the body due to oxidative stress) would be compromised. Therefore, in order to develop an appropriate indicator system for the determination of CAs, it is important that the proposed approach prevents the oxidation of the catechol fragment of DA, NA, and AD, i.e., ensures the stabilization of their complexes with copper ions.

### 3.2. Complexes of Cu(II) Ions and Bispidine-Based Bis-Azole Ligands

**Figure 2:**
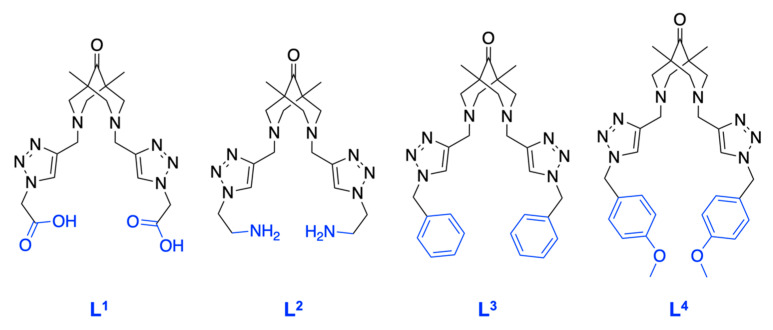
Structures of the bispidine-based bis-azole ligands used in the work. L^1^·2TFA–2,2′-(((1,5-dimethyl-9-oxo-3,7-diazabicyclo[3.3.1]nonane-3,7-diyl)bis(methylene))bis(1H-1,2,3-triazole-4,1-diyl))diacetic acid. L^2^·3.3TFA–3,7-bis((1-(2-aminoethyl)-1H-1,2,3-triazol-4-yl)methyl)-1,5-dimethyl-3,7-diazabicyclo-[3.3.1]nonan-9-one. L^3^–3,7-bis((1-benzyl-1H-1,2,3-triazol-4-yl)methyl)-1,5-dimethyl-3,7-diazabicyclo[3.3.1]nonan-9-one. L^4^–3,7-bis((1-(4-methoxybenzyl)-1H-1,2,3-triazol-4-yl)methyl)-1,5-dimethyl-3,7-diazabicyclo-[3.3.1]nonan-9-one.

In accordance with the classification of vibrational transitions and the selection rules for SERS spectra of molecules with sufficiently high symmetry, the most enhanced lines are the lines due to totally symmetric vibrations that transform according to the unit irreducible representation. In addition, the appearance of forbidden lines associated with antisymmetric vibrations for centrally symmetric molecules can be observed in the SERS spectra. Thus, we proposed the determination of CAs with symmetric polydentate ligands of the bispidine-based bis-azole class (Figure 2) in the presence of Cu(II) ions. As such ligands, we synthesized a set of bispidine-based bis-azoles: L^1^, L^2^, L^3^, and L^4^, which possess similar moiety, differing only in the substituents.

**Figure 3:**
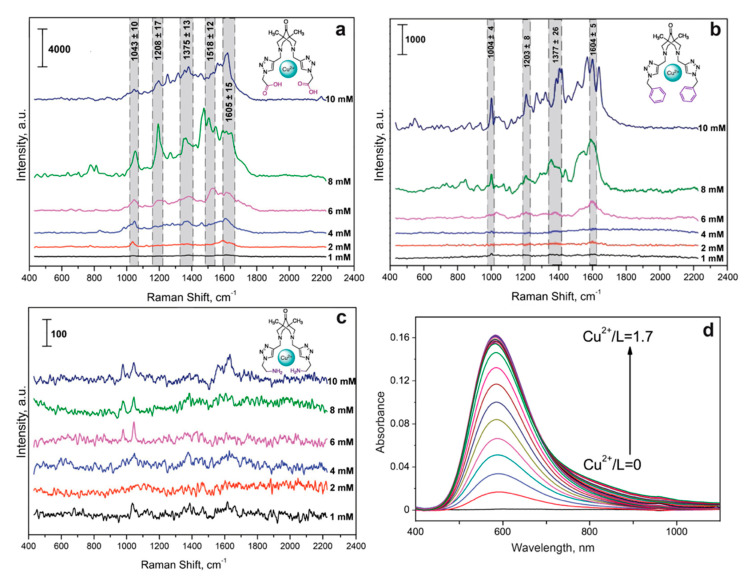
SERS spectra of the bispidine-based bis-azole ligands L^1^ (**a**), L^2^ (**b**), and L^3^ (**d**) with Cu(II) ions at different concentrations. Spectra were measured on the silver nanostructured surface coated with the chitosan layer at 633 nm laser wavelength. (**c**) Typical UV-vis spectra of the triazole ligand L^3^ with Cu(II) ions at the increasing L^3^ concentration.

We studied the formation of complexes between the Cu(II) cation and the bispidine-based bis-azole ligands used, investigated their stability and established the stoichiometry of the resulting complexes (see Supplementary Information File). To do this, each ligand was spectrophotometrically titrated (1 mM) with a Cu(ClO_4_)_2_ solution at 7.5 mM concentration (Figure 3d). The stability constants were calculated using the Bouguer-Lambert-Beer law and the balance of acting masses. To determine the molar absorption coefficients of the resulting complexes, we assumed that at the initial stage of complex formation, the equilibrium concentration of the complex was equal to the concentration of copper cations in the solution. Then, according to the constructed graph of the dependence of the optical density of the solution on the concentration of Cu(II) cations, the molar absorption coefficient was calculated. Based on the data obtained, the true equilibrium concentrations of the ligand and Cu^2+^ and the stability constant of each complex were determined using the relation $K=\frac{\left[Cu_{x}L_{y}\right]}{{\left[Cu\right]}^{x}{\left[L\right]}^{y}}$. As shown in Table 1, the complexes had a preferrable stoichiometry of 1:1.

Importantly, λ_max_ of the obtained complexes were in the visible wavelength range and closer to 633 nm laser (Table 1) compared to absorbance of CAs [43]. The measured stability constants demonstrated that more stable CuL complexes were formed between the Cu(II) cation and L^3^, as with the most nonpolar compound. For the most stable complex, CuL^3^, it was possible to obtain a solid sample and take an X-ray spectrum, confirming the 1:1 structure stoichiometry. For L^1^, a 1:1 complex could not be obtained, which was presumably due to the excessive polarity of the substituent. Based on the obtained data, a 1:1:1 complex was chosen for further determination of CA with various bispidine-based bis-azoles.

We first studied the intrinsic spectra of Cu(II) with the synthesized bispidine-based bis-azole ligands, which could cause a background in SERS spectra for CA detection. Thus, the optimal concentrations of the indicator system were selected by varying the Cu(II) and each of the considered ligands concentrations in the corresponding solvents from Table 1 in the range of 1–10 mM. The complex formation for L^1^ with copper ions on the SERS surface resulted in the significant interfering background signals (Figure 3a). This made determination of catecholamines in the corresponding ternary complexes challenging. Most likely, this was related to the additional interaction of –COOH groups of L^1^ with –NH_2_ groups of chitosan deposited onto silver nanoparticles. Thus, the indicator system with Cu(II) and L^1^ was not further considered for sensitive determination of CAs. For L^3^, its complex formation with copper ions also contributed to the additional spectral background (Figure 3b) due to the intrinsic intense characteristic vibrations of the phenyl groups in L^3^ structure. The SERS spectra shown in Figure 3 demonstrated that L^2^ caused the least background, and the ligands could be used at 1, 4, and 10 mM concentrations for L^1^, L^3^, and L^2^, respectively. L^3^ was advantageous as it showed higher SERS intensities, while L^2^ provided lower spectral background. Thus, L^3^ and L^2^ were chosen for further development of approach for CA detection.

### 3.3. Complexes of Catecholamines with Cu(II) Ions and Bispidine-Based Bis-Azole Ligands

**Figure 4:**
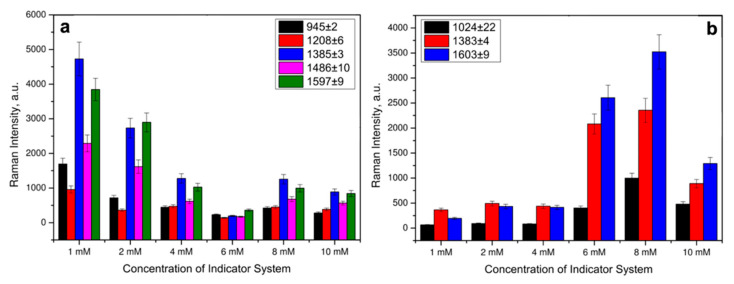
Raman intensities of characteristic DA bands in the SERS spectra at different concentrations of Cu(II) and ligands for: (**a**) L^3^ and (**b**) L^2^. Error bars are shown for *n* = 20, *p* = 0.95.

To identify CA by SERS, the optimal concentration of the indicator system was selected by varying the Cu(II) and each of the considered ligands concentrations in the corresponding solvents from Table 1 in the range of 1–10 mM at a constant concentration of DA, as a model catecholamine. The SERS spectra were recorded with a 633 nm laser wavelength and an extinction time of 10 s. Raman spectra of CAs have been preliminary modeled with density functional theory (DFT) (see Supplementary Information file). Strong modes of CA agreed well with experimental SERS of CA complexes. The SERS spectra in Figure 4a showed that even at an indicator system concentration of 1 mM, intense signals were observed in the SERS spectra for the DA complex with L^3^ and Cu(II) ions. The indicator system with Cu(II) and L^2^ demonstrated stronger SERS signals of DA at 8 mM concentration based on the majority of Raman peaks (Figure 4b). The difference in the optimal concentrations might be connected to the differences in chemical structure of the studied ligands. From the SERS spectra shown in Figure 3, carboxyl and phenyl groups of L^1^ and L^3^, respectively, demonstrated more effective coordination to the AgNPs surface, compared to amino groups of L^2^.

**Figure 5:**
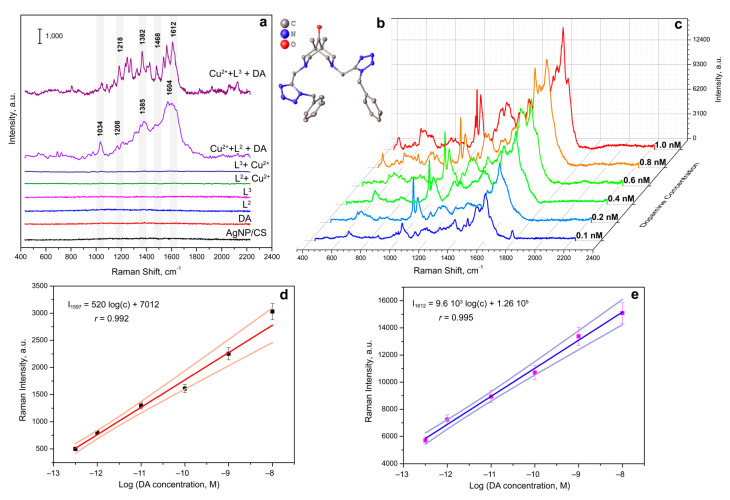
(**a**) Typical SERS spectra of the blank non-modified SERS substrate, DA (0.1 mM), L^2^ (8 mM), L^3^ (1 mM), the indicator system: Cu(II) ions with L^2^ (8 mM), the indicator system: Cu(II) ions with L^3^ (1 mM), DA (0.1 mM) applied onto the SERS substrate modified with Cu(II) ions and L^2^ (8 mM), and DA (0.1 mM) applied onto the SERS substrate modified with Cu(II) ions and L^3^ (1 mM). (**b**) Single-crystal structure of the bispidine-based bis-azole ligand L^3^. (**c**) SERS spectra of various DA concentrations applied onto the SERS substrate modified with Cu(II) ions and L^3^ (1 mM). All SERS spectra were measured on the silver nanostructured surface coated with the chitosan layer at the resonant 633 nm laser wavelength. (**d**) Calibration curve for DA determination with Cu(II) ions and L^2^ (8 mM) based on Raman intensity of 1597 cm^−1^ peak. Pale orange lines demonstrate confidence corridor with *p* = 0.95. (**e**) Calibration curve for DA determination with Cu(II) ions and L^3^ (1 mM) based on Raman intensity of 1612 cm^–1^ peak. Pale blue lines demonstrate confidence corridor with *p* = 0.95.

**Figure 6:**
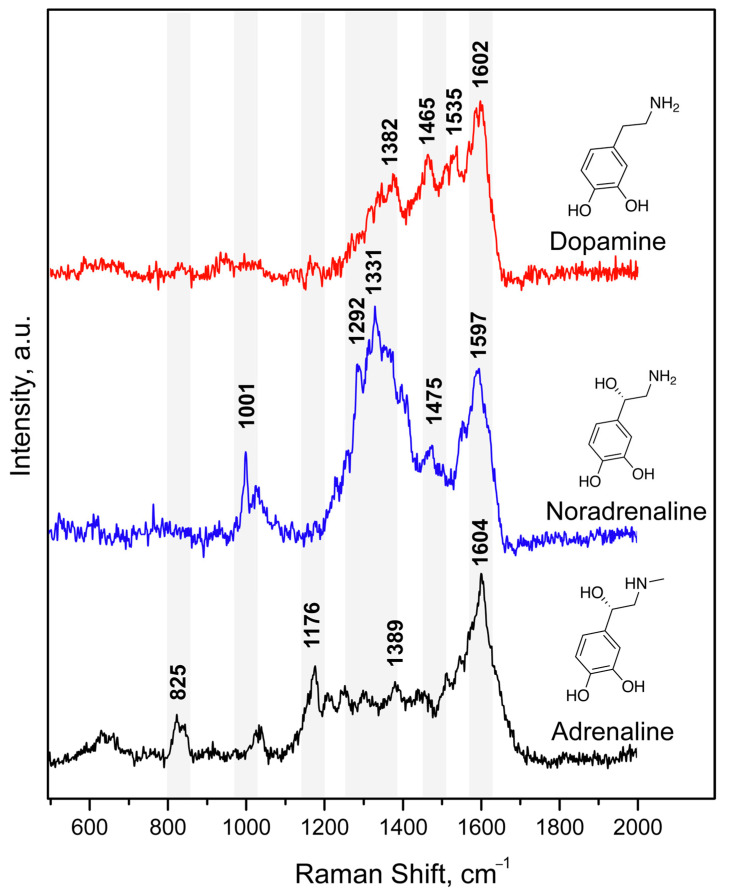
SERS spectra of the DA (0.1 mM), NA (0.1 mM), and AD (0.1 mM) applied onto the SERS substrate modified with Cu(II) ions and L^3^ (1 mM) at 633 nm excitation laser wavelength.

With using identical experimental conditions, areas of the SERS-sensing surface, concentrations and volumes of solutions of the Cu(II):bispidine-based bis-azole components applied to the surface, and Raman microscope settings, we obtained the calibration curves for CAs (Figure 5). Notably, the L^3^ ligand with phenyl groups demonstrated sharper SERS peaks as well as higher sensitivity compared to the indicator system with amino groups in L^2^. This could be explained by NH_2_-groups in L^2^ structure also interacting with the AgNP-based surface resulting in the presence of differently oriented DA:Cu(II):L^2^ complexes on the SERS substrate surface. Figure 5c shows the SERS spectra of DA obtained on the developed SERS-active surface at various concentrations of DA in the reaction mixture (the indicator system is L^3^:Cu(II) ions at 1 mM concentration). The linear trend for DA determination was observed at a 3.2 × 10^−12^–1 × 10^−8^ M concentration range. Normal values of catecholamines in blood plasma were reported to be 4 × 10^−11^–4.5 × 10^−9^ M [46], 4.5 × 10^−10^–2.49 × 10^−9^ M [47], and 2 × 10^−11^–4.67 × 10^−10^ M [47] for DA, NA, and AD, respectively. Thus, the proposed approach demonstrated promise for rapid multiplex determination of catecholamines: dopamine, adrenaline, and noradrenaline which could be applied for “point-of-care” analysis (Figure 6).

## 4. Conclusions

Herein, we propose an approach for catecholamine detection based on trapping them into colored stable complexes on the SERS-active surface. The MIRRACLE (Molecular Immobilization and Resonant Raman Amplification by Complex-Loaded Enhancers) methodology allowed for sensitive determination of dopamine in 3.2 × 10^−12^–1 × 10^−8^ M concentration range. The determination of catecholamines in complexes with Cu(II) and bispidine-based bis-azole ligands on a nanostructured silver surface coated with a thin layer of chitosan by SERS allowed the selective determination of dopamine, adrenaline, and noradrenaline. Four synthesized bispidine-based bis-azole ligands served as pliers in the ternary complex with Cu(II) ions and catecholamines. The limits of detection of catecholamines using the developed method make it possible to determine them at the level of picomolar concentrations, which corresponds to the reference values in the blood plasma of healthy people. Therefore, the use of the proposed technique provides a potential opportunity to determine catecholamine neurotransmitters in biological fluids using SERS spectroscopy, specifically for diagnosis of neuroendocrine disorders. Further prospects in this area lie in the improvement and optimization of methods for the multiplexed determination of the largest possible set of molecules–markers of neurotransmitter metabolism disorders, as well as the subsequent detection of the correlation of neuroendocrine and neurodegenerative diseases with concentrations and their ratios in biological fluids with the possibility of non-invasive diagnostics.

## Figures and Tables

**Table 1:** Stability constants of complexes of bispidine-based bis-azoles with Cu(II) with various stoichiometry. Stability constants were calculated based on the UV-vis measurements using the Bouguer-Lambert-Beer law and the mass action law (see Supplementary Information file).

Ligand, Solvent	L^1^, H_2_O	L^2^, EtOH	L^3^, MeCN	L^4^, MeCN
lgK(CuL_2_)	–*	–*	8.3 ± 0.5	–*
lgK(CuL)	–*	4.0 ± 0.1	6.0 ± 0.5	5.2 ± 0.3
lgK(Cu_2_L_3_)	23.0 ± 0.3 (pH 4.0)	–*	–*	–*
λ_max_, nm	577	595	583	577

* the complexes of this stoichiometry did not form.

## Data Availability

Not applicable.

## Supplementary Materials

The following supporting information can be downloaded at: https://www.mdpi.com/article/10.3390/bios13010124/s1. Figure S1: UV-vis spectra of 5 mM solutions of 8a–AD, 8b–DA and their corresponding complexes with: 1–Cu(II), 2–Al(III), 3–Fe(II), 4–Co(II), 5–Fe(III), 6–Ni(II), 7–Mg(II). Figure S2: UV-vis spectra of DA and Cu(II) equimolar mixture in time 0–15 min. Table S1: Raman bands theoretically calculated, published in the literature, and experimentally observed on the AgNP-based substrate for [Cu(semiquinone)_3_]^−^ (λ_ex_ 632.8 nm). Figure S3: UV-vis spectra for spectrophotometric titration of L^4^ with Cu^2+^ solution in (a) 100% MeCN; (b) 50% MeCN–50% H_2_O. Figure S4: Spectrophotometric titration of L^4^ with Cu^2+^ solution in (a) 100% MeCN (λ_abs_ = 577 nm); (b) 50% MeCN–50% H_2_O (λ_abs_ = 583 nm). Figure S5: (a) UV-vis spectra for spectrophotometric titration of Cu^2+^ with L^4^ solution in 50% MeCN–50% H_2_O. (b) Spectrophotometric titration of Cu^2+^ with L^4^ solution in 50% MeCN–50% H_2_O (λ_abs_ = 583 nm). Figure S6: UV-vis spectra for spectrophotometric titration of L^3^ with Cu^2+^ solution in (a) 100% MeCN; (b) 50% MeCN–50% H_2_O. Spectrophotometric titration of L^3^ with Cu^2+^ solution in (c) 100% MeCN (λ_abs_ = 577 nm and λ_abs_ = 697 nm); (b) 50% MeCN–50% H_2_O (λ_abs_ = 583 nm). Figure S7: (a) UV-vis spectra for spectrophotometric titration of Cu^2+^ with L^3^ solution in 50% MeCN–50% H_2_O. (b) Spectrophotometric titration of Cu^2+^ with L^3^ solution in 50% MeCN–50% H_2_O (λ_abs_ = 583 nm). Figure S8: (a) UV-vis spectra for spectrophotometric titration of Cu^2+^ with L^4^ solution in 100% MeCN. Figure S9: Spectrophotometric titration of L^1^ with Cu^2+^ solution in H_2_O (λ_abs_ = 577 nm). Table S2: The calculated values of the constants of complex formation of copper with water-insoluble bispidine-type ligands. Table S3: The protonation constants for L^1^ in H_2_O (μ = 0.1 M) at 25 °C. Figure S10: (a) Potentiometric titration of L^1^ (1 mM) in H_2_O in the presence of HClO_4_ (4 mM), μ = 0.1 Μ ΚΝO_3_ at 25 °C with NaOH solution (0.1 M). (b) Distribution of protonated and deprotonated forms of L^1^ (1 mM) in solution depending on pH. Table S4: The stability constants of complexes of L^1^ with cations Cu^2+^ and hydroxocomplexes Cu^2+^ in an aqueous solution (μ = 0.1 M) at 25 °C. Figure S11: (a) Potentiometric titration of L^1^ and L^1^ in the presence of Cu^2+^ (Cu^2+^ + L) in H_2_O (μ = 0.1 Μ ΚΝO_3_) at 25 °C. (b) Distribution of different forms of L^1^ (1 mM) in the presence of Cu(II) perchlorate (1 mM) depending on pH. Figure S12: Typical SEM images of a proposed silver nanoparticle sensor surface (a) coated with a silver chitosan film of a silver nanoparticle surface (d) and its cross section (c); optical photograph of a silver nanostructured surface consisting of overlapping “coffee rings” (b). Figure S13: SERS spectra of various AD concentrations applied onto the SERS substrate modified with Cu(II) ions and L^3^ (1 mM). All SERS spectra were measured on the silver nanostructured surface coated with the chitosan layer at the resonant 633 nm laser wavelength. Figure S14: SERS spectra of various NA concentrations applied onto the SERS substrate modified with Cu(II) ions and L^3^ (1 mM). All SERS spectra were measured on the silver nanostructured surface coated with the chitosan layer at the resonant 633 nm laser wavelength. Figure S15: Optimized geometry for dopamine (DA) in “Q-Chem” with PBE0 method and 6-31G* basis. Figure S16: Calculated normalized Raman spectrum for dopamine (DA) in “Q-Chem” with PBE0 method and 6-31G* basis (Lorentzian). Table S5: Calculated normalized Raman shifts with intensities for dopamine (DA) in “Q-Chem” with PBE0 method and 6-31G* basis (Lorentzian). Figure S17: Optimized geometry for noradrenaline (NA) in “Q-Chem” with PBE0 method and 6-31G* basis. Figure S18: Calculated normalized Raman spectrum for noradrenaline (NA) in “Q-Chem” with PBE0 method and 6-31G* basis (Lorentzian). Table S6: Calculated normalized Raman shifts with intensities for noradrenaline (NA) in “Q-Chem” with PBE0 method and 6-31G* basis (Lorentzian). Figure S19: Optimized geometry for adrenaline (AD) in “Q-Chem” with PBE0 method and 6-31G* basis. Figure S20: Calculated normalized Raman spectrum for adrenaline (AD) in “Q-Chem” with PBE0 method and 6-31G* basis (Lorentzian). Table S7: Calculated normalized Raman shifts with intensities for adrenaline (AD) in “Q-Chem” with PBE0 method and 6-31G* basis (Lorentzian).
