# Supplementary material for: Plier Ligands for Trapping Neurotransmitters into Complexes for Sensitive Analysis by SERS Spectroscopy
Source: Biosensors (Basel). 2023 Jan 11;13(1):124. doi: 10.3390/bios13010124 (PMC9856153; doi:10.3390/bios13010124)
Supplement: Supplementary file 1 [file biosensors-13-00124-s001.zip › biosensors-2126761-supplementary.pdf]

Supplementary Information

Plier Ligands for Trapping Neurotransmitters into Complexes for Sensitive Analysis by SERS Spectroscopy

Olga E. Eremina,[a] Olesya O. Kapitanova,[a] Alexei V. Medved'ko,[b] Alexandra S. Zelenetskaya,[a] Bayirta V. Egorova,[a] Tatyana N. Shekhovtsova,[a*] Sergey Z. Vatsadze,[b] and Irina A. Veselova[a]

[a.] Chemistry Department, Moscow State University, Moscow 119991, Russia

[b.] N.D.Zelinsky Institute of Organic Chemistry, Russian Academy of Sciences, Moscow 119991, Russia

*corresponding author: tnshekh@yandex.ru

Table of Content

I. Catecholamine complexes with metal ions..S2

II. Formation of triazole complexes with Cu(II) S3

III. SERS sensor S10

IV. Theoretical Raman spectra of Catecholamines..S12

I. Catecholamine Complexes with Metal Ions

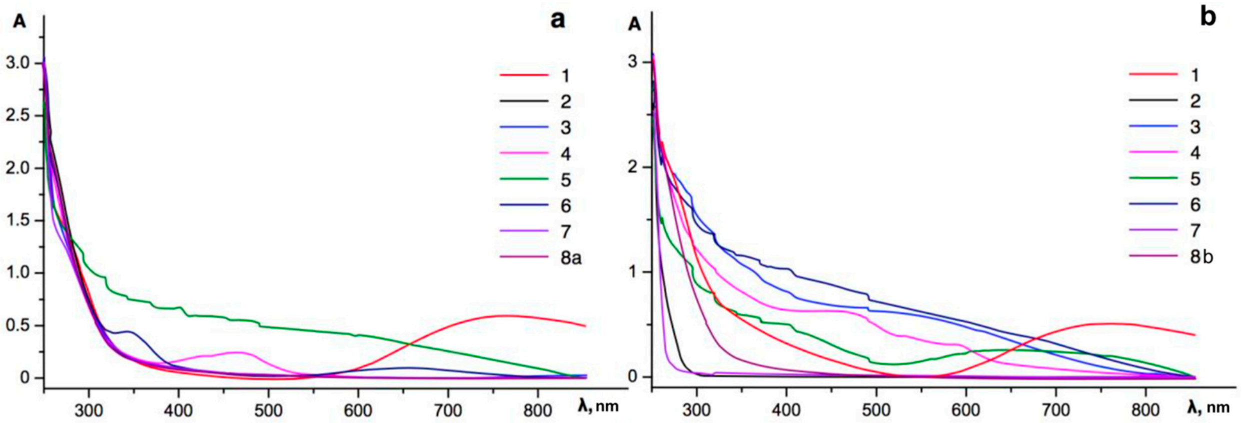

Figure S1. UV-vis spectra of 5 mM solutions of 8a – AD, 8b – DA and their corresponding complexes with: 1 – Cu(II), 2 – Al(III), 3 – Fe(II), 4 – Co(II), 5 – Fe(III), 6 – Ni(II), 7 – Mg(II).

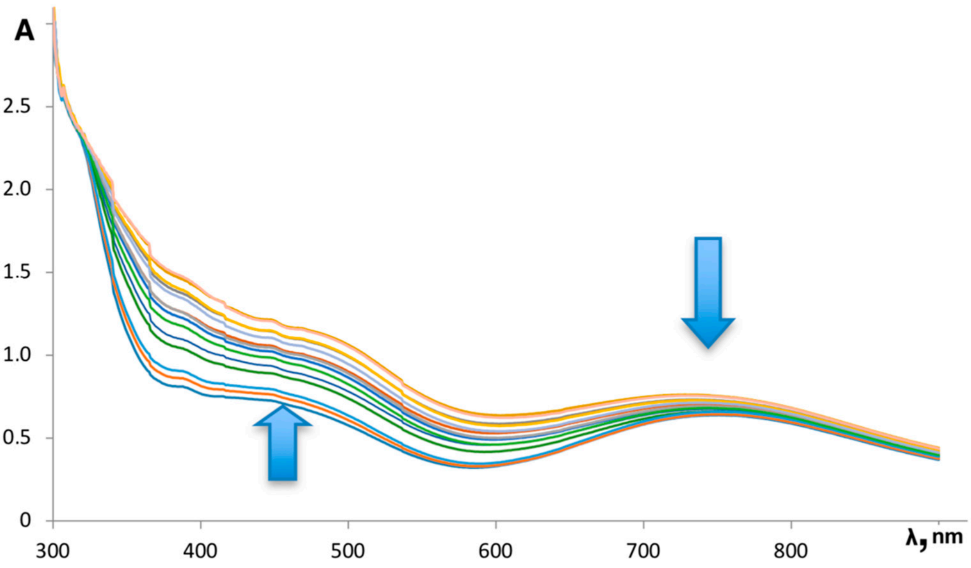

Figure S2. UV-vis spectra of DA and Cu(II) equimolar mixture in time 0 – 15 min.

Table S1. Raman bands theoretically calculated, published in the literature, and experimentally observed on the AgNP-based substrate for $[Cu(semiquinone)_3]^-$ (λ_{ex} 632.8 nm).

Vibrational Modes	Strength	Raman Shift, cm^{-1}		
		Theoretical	Literature [1]	Experimental
ν (C–C)	v weak	1690	1675	–
ν $(C–C)_{ring}$	medium	1654	1590	1596 ± 8
ν (C–C)	strong	1545	–	1530 ± 8
ν $(C_1–C_2)$ + ν (C=O)	v strong	1409	1383	1382 ± 2
δ (C–H) + ν (C=O)	v weak	–	1255	1209 ± 5
δ (C–H)	medium	1118	1027	–
δ (C–H)	medium	1134	1016	1134 ± 4
δ (C–H)	medium	–	991	–
ν (C–C) + ν (C=O)	medium	976	950	948 ± 2
δ (C–H)	medium	922	915	–
ν $(M)_{ring}$	–	580	–	584 ± 2
ν $(M)_{ring}$	–	538	–	–
ν $(M)_{ring}$	v weak	–	490	497 ± 4
ν (Cu–ring)	medium	387	379	–

II. Formation of triazole complexes with Cu(II)

The study of complexation process for **L⁴** was carried out in MeCN and MeCN:H_2O (1:1) for comparison stability of complexes being formed with and without H_2O (Figures S3). Based on the obtained data, the stoichiometry of the obtained complexes was determined by the molar ratio method.

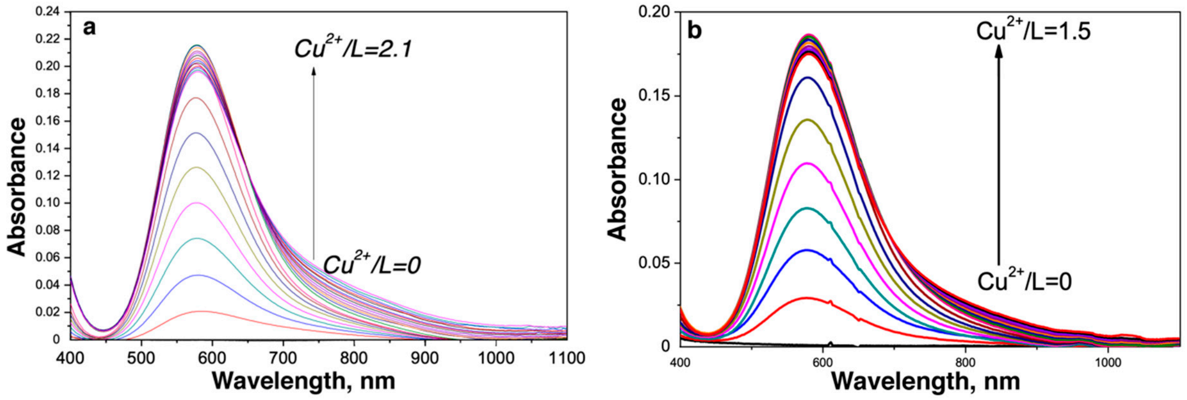

Figure S3. UV-vis spectra for spectrophotometric titration of **L⁴** with Cu^{2+} solution in (**a**) 100% MeCN; (**b**) 50% MeCN – 50% H_2O.

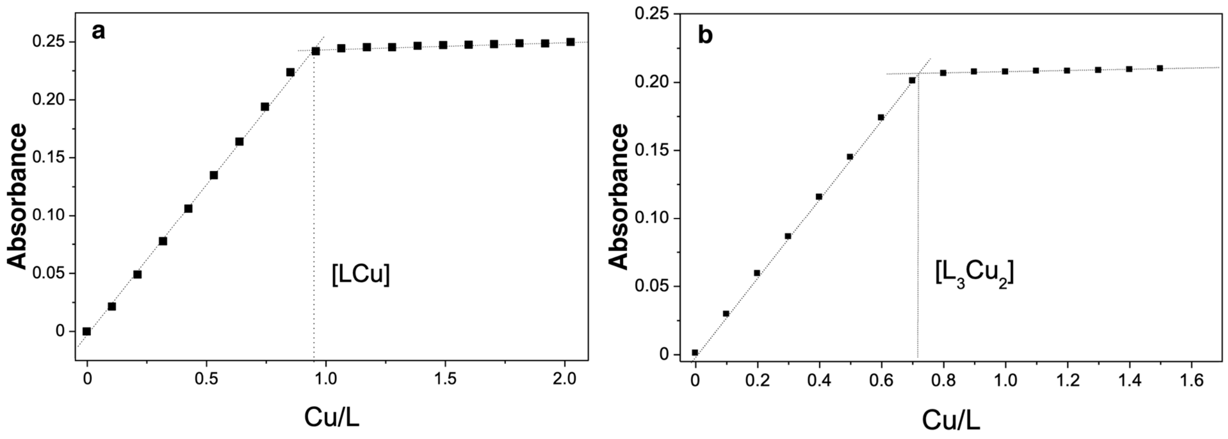

Figure S4. Spectrophotometric titration of **L⁴** with Cu^{2+} solution in (**a**) 100% MeCN (λ_{abs} = 577 nm); (**b**) 50% MeCN – 50% H_2O (λ_{abs} = 583 nm).

For **L⁴**, the formation of a complex with the ratio Cu:L = 1:1 (λ_{abs} = 577 nm) in acetonitrile and the complex Cu:L = 2:3 (λ_{abs} = 583 nm) in a mixture of MeCN:H_2O = 1:1. Titration was carried out in the opposite direction, that is, starting with a lack of ligand: the L4 solution was added to the cuvette containing copper cations. The stoichiometry of Cu_2L_3 complex was confirmed (Figures S4). However, in this case, under the conditions of a lack of ligand, the L:Cu = 1:1 complex is first formed, and with the further addition of the ligand to the copper solution, the LCu

complex becomes L_3Cu_2 complex. Both complexes are characterized by absorption at 583 nm (Figures S5).

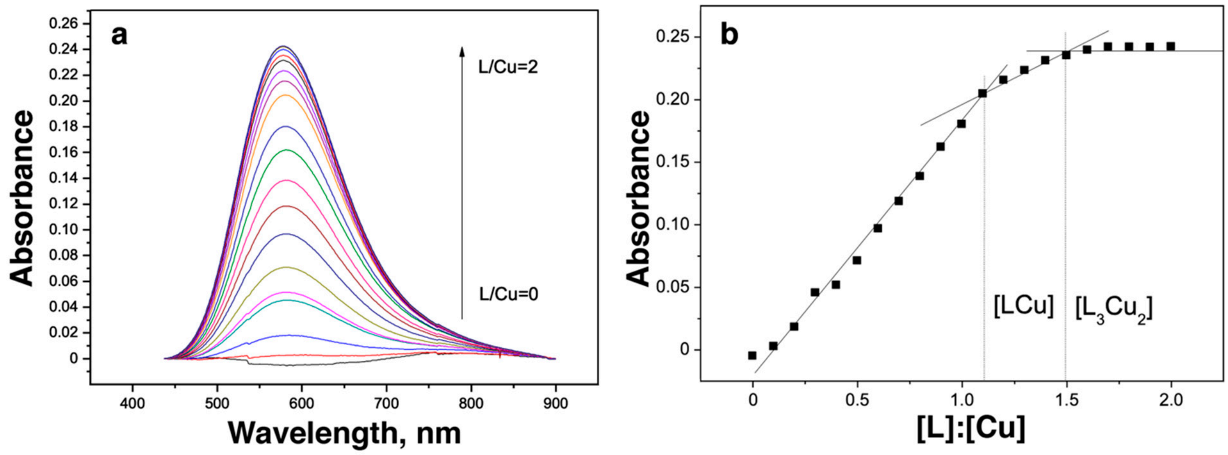

Figure S5. (**a**) UV-vis spectra for spectrophotometric titration of Cu^{2+} with **L**[4] solution in 50% MeCN – 50% H_2O. (**b**) Spectrophotometric titration of Cu^{2+} with **L**[4] solution in 50% MeCN – 50% H_2O (λ_{abs} = 583 nm).

L[3] exhibits properties like **L**[4], and forms LCu complexes with absorption at the same wavelength. In MeCN, with excess of ligand, the intermediate L_2Cu complex is first formed (λ_{abs} = 697 nm), which, with the subsequent addition of copper cations, passes into the LCu complex with 1:1 stoichiometry and absorption at λ_{abs} = 577 nm 577 nm (Figure S6). However, in a mixture of acetonitrile and water, such a change in the spectrum is not observed (Figure S6), only a regular increase in absorption at 583 nm (Figure S7). In the latter case, the stoichiometry complex Cu:L = 2:3 is formed (Figure S8).

Upon titration of copper cations in the opposite direction with **L**[3] solution in 50% MeCN – 50% H_2O, LCu complex is formed (Figure S8), which, unlike the complex with **L**[4], does not transform into complexes with a different stoichiometry upon subsequent addition ligand in the presence of copper.

The complex of **L**[1] with copper absorbs only at 577 nm, as Cu:L = 1:1 and 2:3 complexes with water-insoluble ligands **L**[4] and **L**[3]. Titration at pH 4.0 and 9.0 shows that 2:3 complexes are formed in both cases.

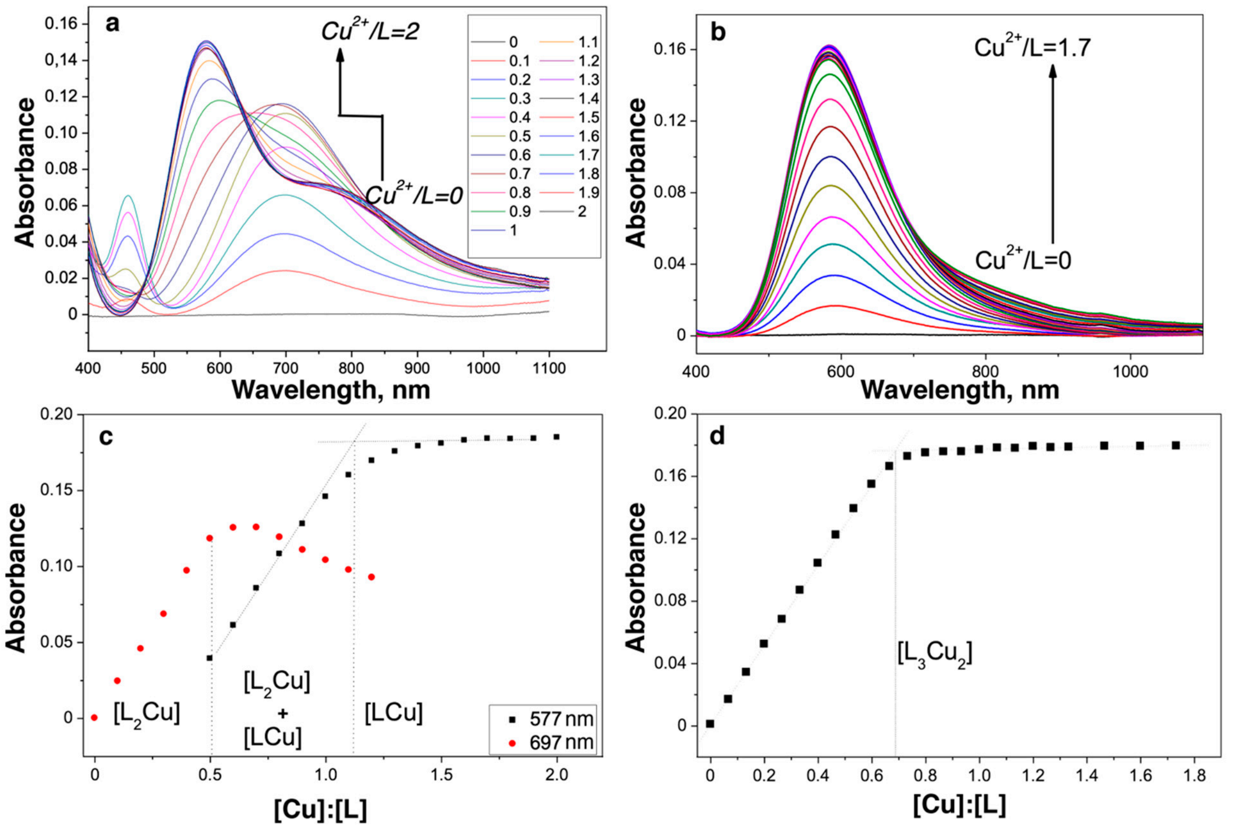

Figure S6. UV-vis spectra for spectrophotometric titration of $\mathbf{L^3}$ with Cu^{2+}solution in (**a**) 100% MeCN; (**b**) 50% MeCN – 50% H_2O. Spectrophotometric titration of $\mathbf{L^3}$ with Cu^{2+} solution in (**c**) 100% MeCN (λ_{abs} = 577 nm and λ_{abs} = 697 nm); (**b**) 50% MeCN – 50% H_2O (λ_{abs} = 583 nm).

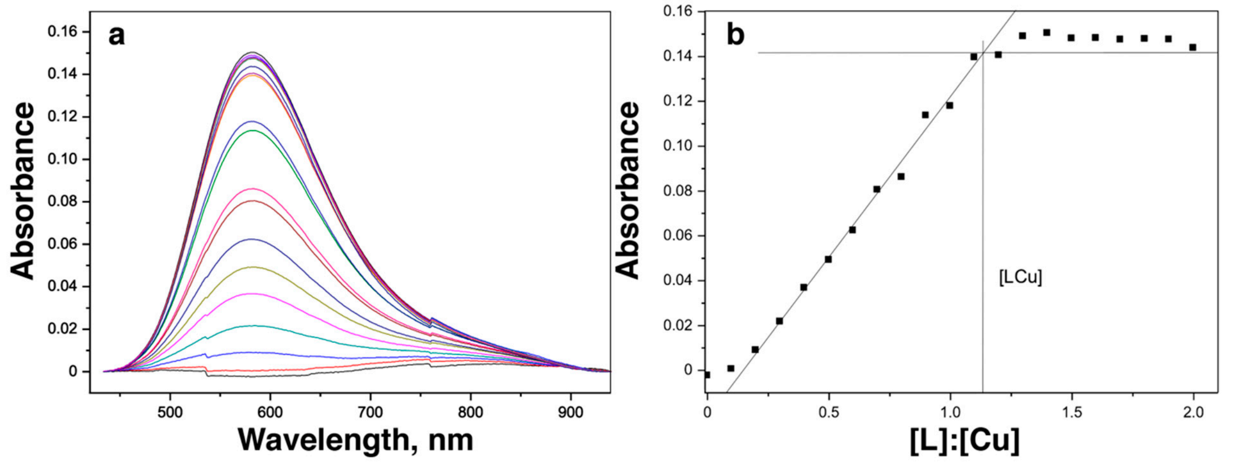

Figure S7. (**a**) UV-vis spectra for spectrophotometric titration of Cu^{2+} with $\mathbf{L^3}$ solution in 50% MeCN – 50% H_2O. (**b**) Spectrophotometric titration of Cu^{2+} with $\mathbf{L^3}$ solution in 50% MeCN – 50% H_2O (λ_{abs} = 583 nm).

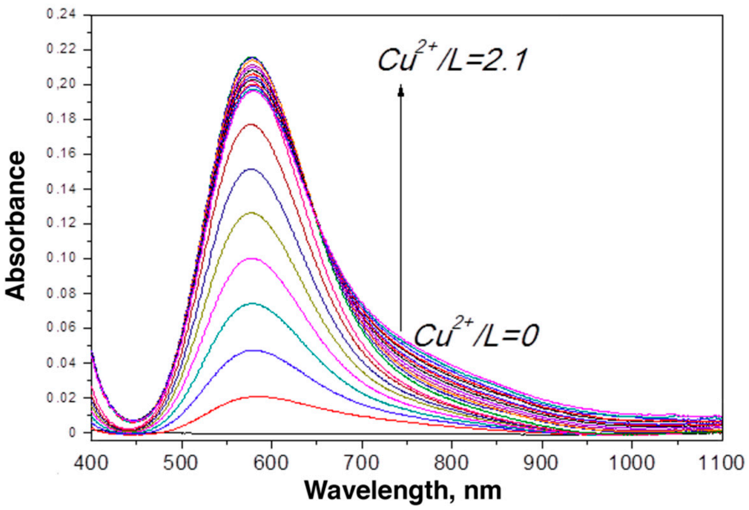

Figure S8. UV-vis spectra for spectrophotometric titration of Cu^{2+} with $\mathbf{L^4}$ solution in 100% MeCN.

The obtained dependences were used for determination of stability constants of copper complexes. All constants were determined using the Bouguer-Lambert-Beer law and mass balance. To determine the extinction coefficient of the resulting complexes, it was assumed that at the initial stage of complex formation, the equilibrium concentration of the complex is equal to the concentration of copper cations in solution. We plotted the dependence of the optical density of the solution on the concentration of copper cations, the extinction coefficient was determined from the trend line, and with its help the equilibrium concentration of the copper complex with the ligand was recalculated at subsequent points.

Based on the data obtained, [L] and $[Cu^{2+}]$ were determined, and then the stability constant of the complex was determined (Table S2) as the ratio of the equilibrium concentration of the complex to the product of the remaining concentrations of the ligand and copper cation in the degree of their stoichiometric coefficients:

$$K_f = \frac{[Cu_xL_y]}{[Cu]^x \cdot [L]^y}$$

The results calculated through direct and reverse titration correlate well with each other (Table S2). In addition, it can be seen that, within the error, lgK values for the CuL complexes in presence of and without H_2O do not differ. The latter may be associated with the formation of the same complex in both environments.

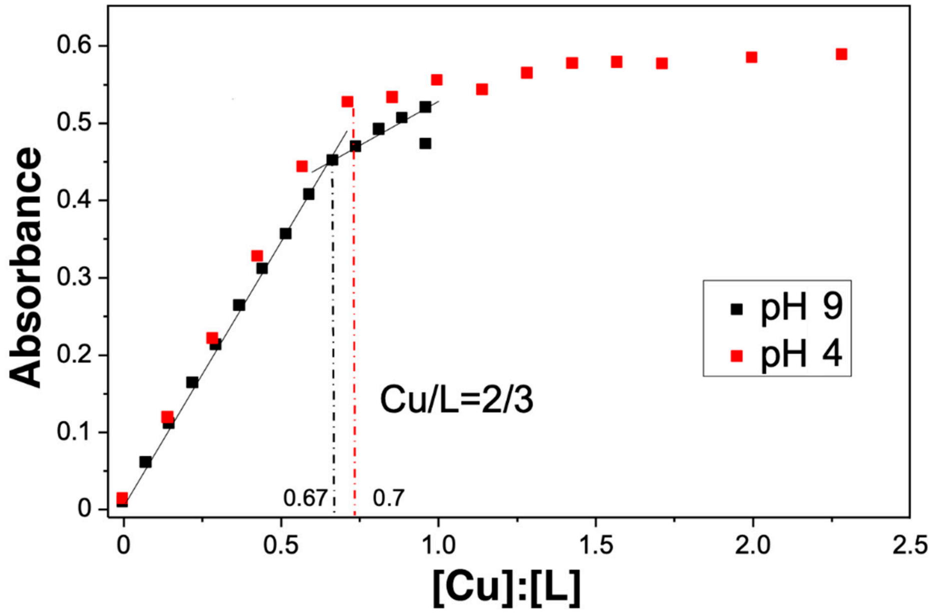

Figure S9. Spectrophotometric titration of **L¹** with Cu^{2+} solution in H_2O (λ_{abs} = 577 nm).

In the case of pH 4.0, the constant is higher compared to the constant at pH 9.0. The obtained value at pH 4.0 correlates to some extent with the results of potentiometry: at low pH 3 – 6 (Figure S9), the monoprotonated complex form with lgK = 23 prevails (both by potentiometry and spectrophotometry). At a higher pH 9.0, a completely deprotonated complex is formed (according to potentiometry, Figure S10), the constant of which is like the constant obtained for **L⁴** and **L⁴** in 50% acetonitrile (Table S2).

Table S2. The calculated values of the constants of complex formation of copper with water-insoluble bispidine-type ligands.

Ligand and conditions	L^1 100% H_2O	L^2 100% EtOH	L^3 100% MeCN	L^3 50% MeCN	L^4 100% MeCN	L^4 50% MeCN
lgK(CuL_2)	–	–	8.3 ± 0.5^A	–	–	–
lgK(CuL)	–	4.0 ± 0.1	6.0 ± 0.5^A	5.2 ± 0.5^B	5.2 ± 0.3^A	5.9 ± 0.5^B
lgK(Cu_2L_3)	23.0 ± 0.3^A (pH 4.0) 17.4 ± 0.5^A (pH 9.0)	–	–	16.4 ± 0.5^A	–	17.3 ± 0.5^A 17.1 ± 0.5^B
λ_{abs}, nm	577	595	577/697	583	577	583

^{A} direct titration: addition of ligand to Cu^{2+} solution.

^{B} reverse titration: addition of Cu^{2+} to ligand solution.

Thus, it can be concluded that the triazole groups affect not only the solubility of the ligands, but also the stoichiometry and stability of the complexes formed. With an increase in the

polarity of the solvent with the addition of water, a tendency is observed to form complexes with a high specific ligand content per cation.

The protonation constants for $\mathbf{L^1}$ were calculated. For this, $\mathbf{L^1}$ was added to the $HClO_4$ solution and then titrated with an alkali solution, measuring the potential of the indicator electrode. The experimental titration curve for $\mathbf{L^1}$, the calculated protonation constants, and the plot of the protonated and non-protonated forms of the ligand in solution versus pH are shown in Figure S10 and Table S3. During titration in an aqueous solution, $\mathbf{L^1}$ was able to release 3 protons from 2 protonated amino groups and one carboxyl.

Table S3. The protonation constants for $\mathbf{L^1}$ in H_2O (μ = 0.1 M) at 25 °C.

**L1**	H^+	Equations for stability constants	lgK	Equilibrium
1	1	K_{LH}=[**L**·H^+]/[**L**]·[H^+]	11.8 ± 0.2	**L**+H^+↔**L·**H^+
1	2	K_{LH2}=[**L**)(H^+)$_2$]/[**L**]·[H^+]^2	21.0 ± 0.2	(**L**)+2H^+↔(**L**)**·**(H^+)$_2$
1	3	K_{LH3}=[**L**·(H^+)$_3$]/[**L**]·[H^+]^3	23.8 ± 0.2	(**L**)+3H^+↔(**L**)**·**(H^+)$_3$

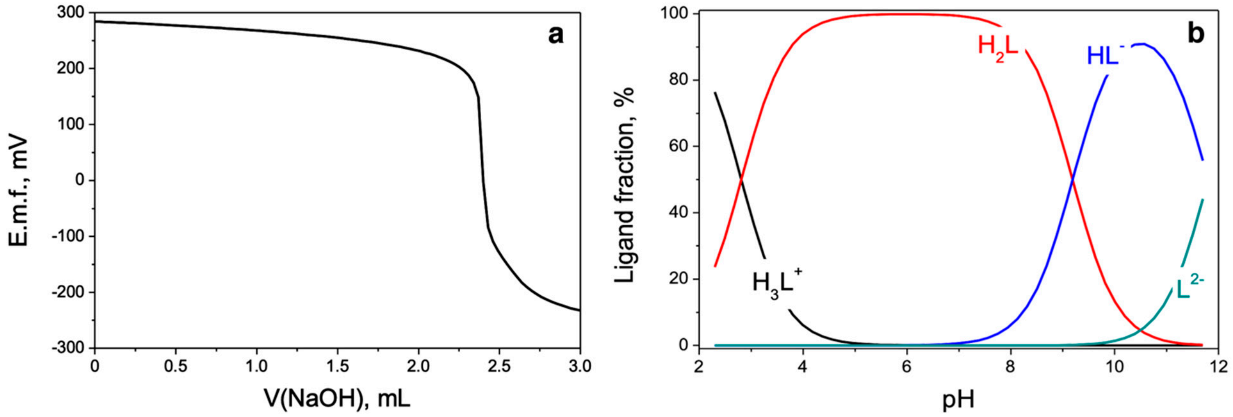

Figure S10. (**a**) Potentiometric titration of $\mathbf{L^1}$ (1 mM) in H_2O in the presence of $HClO_4$ (4 mM), μ = 0.1 M KNO_3 at 25 °C with NaOH solution (0.1 M). (**b**) Distribution of protonated and deprotonated forms of $\mathbf{L^1}$ (1 mM) in solution depending on pH.

The complexation of ligand $\mathbf{L^1}$ with a copper(II) cation was studied in the range of pH 2 – 12. The formation of complexes of the composition (H**L**)(Cu^{2+}), (**L**)(Cu^{2+}), (L)(Cu^{2+})(OH^-), the previously calculated stability constants of the complexes (**L**)·H^+, (**L**)·(H^+)$_2$, (**L**)·(H^+)$_3$ constants of hydrolysis of copper(II) cations and water dissociation. The calculated values of the constants and published data on the stability of hydroxocomplexes of Cu(II) ions are presented in Table S4.

Table S4. The stability constants of complexes of **L**[1] with cations Cu^{2+} and hydroxocomplexes Cu^{2+} in an aqueous solution (μ = 0.1 M) at 25 ºC.

**L**	Cu^{2+}	H^+	lgβ	Equilibrium
1	1	1	23.17 ± 0.02	H^++(**L**)+Cu^{2+}↔(H**L**)·(Cu^{2+})
1	1	0	16.03 ± 0.06	(**L**)+Cu^{2+}↔(**L**)·(Cu^{2+})
1	1	–1	4.69 ± 0.06	(**L**)+Cu^{2+}+H_2O↔(**L**)·(Cu^{2+})·(OH^-)+H^+
0	1	–1	–7.96	$Cu^{2+} + H_2O \leftrightarrow (CuOH)^+ + H^+$
0	1	–2	–16.24	$Cu^{2+} + 2H_2O \leftrightarrow Cu(OH)_2 + 2H^+$
0	1	–3	–26.7	$Cu^{2+} + 3H_2O \leftrightarrow Cu(OH)_3^- + 3H^+$
0	1	–4	–39.6	$Cu^{2+} + 4H_2O \leftrightarrow (Cu(OH)_4)^{2-} + 4H^+$
0	2	–1	–6.7	$2Cu^{2+} + H_2O \leftrightarrow (Cu_2OH)^{3+} + H^+$
0	2	–2	–10.35	$2Cu^{2+} + H_2O \leftrightarrow (Cu_2(OH))^{2+} + 2H^+$
0	3	–4	–21.1	$3Cu^{2+} + 4H_2O \leftrightarrow (Cu_3(OH)_4)^{2+} + 4H^+$

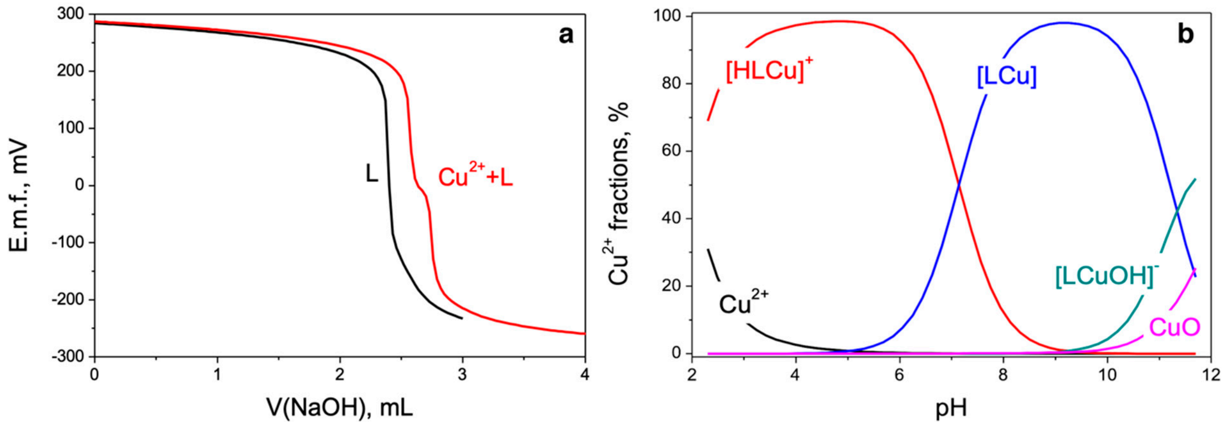

Figure S11. (**a**) Potentiometric titration of **L**[1] and **L**[1] in the presence of Cu^{2+} (Cu^{2+} + L) in H_2O (μ = 0.1 M KNO_3) at 25 ºC. (**b**) Distribution of different forms of **L**[1] (1 mM) in the presence of copper(II) perchlorate (1 mM) depending on pH.

III. SERS Sensor

Highly sensitive and selective determination of catecholamines in various biological samples for the diagnosis of neuroendocrine tumors and neurodegenerative dementia remains an urgent problem in modern chemical analysis. One of the most promising, relatively new and rapidly developing methods around the world is spectroscopy of surface-enhanced, or giant, Raman scattering (SERS). The phenomenon of a sharp increase in the intensity of the Raman signal ($10^4 – 10^{12}$ times) is based on the effect of surface plasmon resonance on a nanostructured surface, as a rule, of noble metals.

We have developed a planar sensor device consisting of silver nanoparticles with a size of 30 – 50 nm that form aggregates (Figure S12) on a thin glass plate. The silver surface was synthesized by pyrolysis of an aerosol of the ammonia complex of silver $[Ag(NH_3)_2]OH$ (1.25 mM):

$$Ag^{+} + OH^{-} \rightarrow Ag^{0} + \frac{1}{4}O_2 + \frac{1}{2}H_2O$$

$$3Ag^{+} + NH_3 + 3\,OH^{-} \rightarrow 3Ag^{0} + \frac{1}{2}N_2 + 3H_2O$$

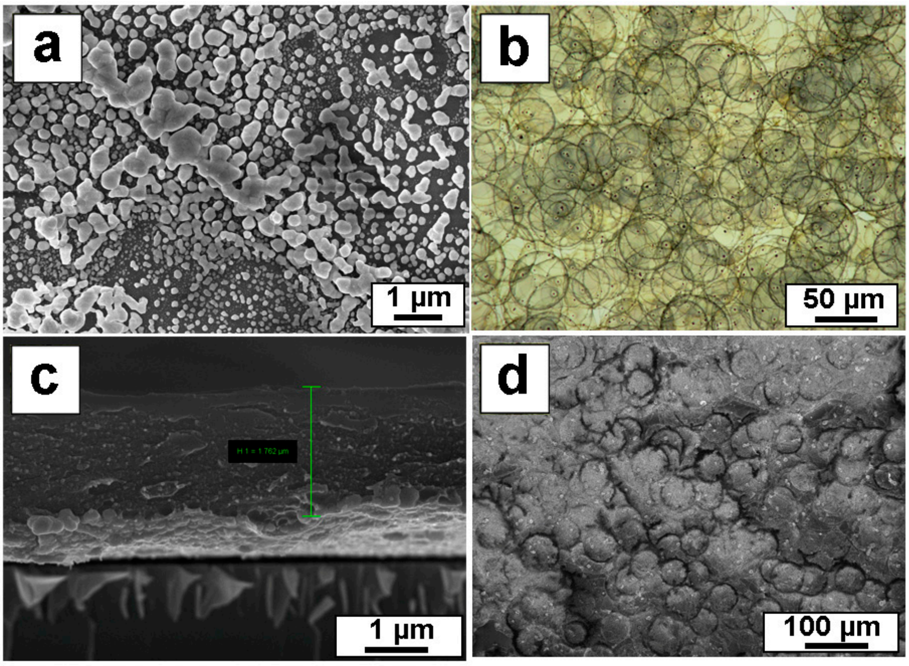

Figure S12. Typical SEM images of a proposed silver nanoparticle sensor surface (**a**) coated with a silver chitosan film of a silver nanoparticle surface (**d**) and its cross section (**c**); optical photograph of a silver nanostructured surface consisting of overlapping “coffee rings” (**b**).

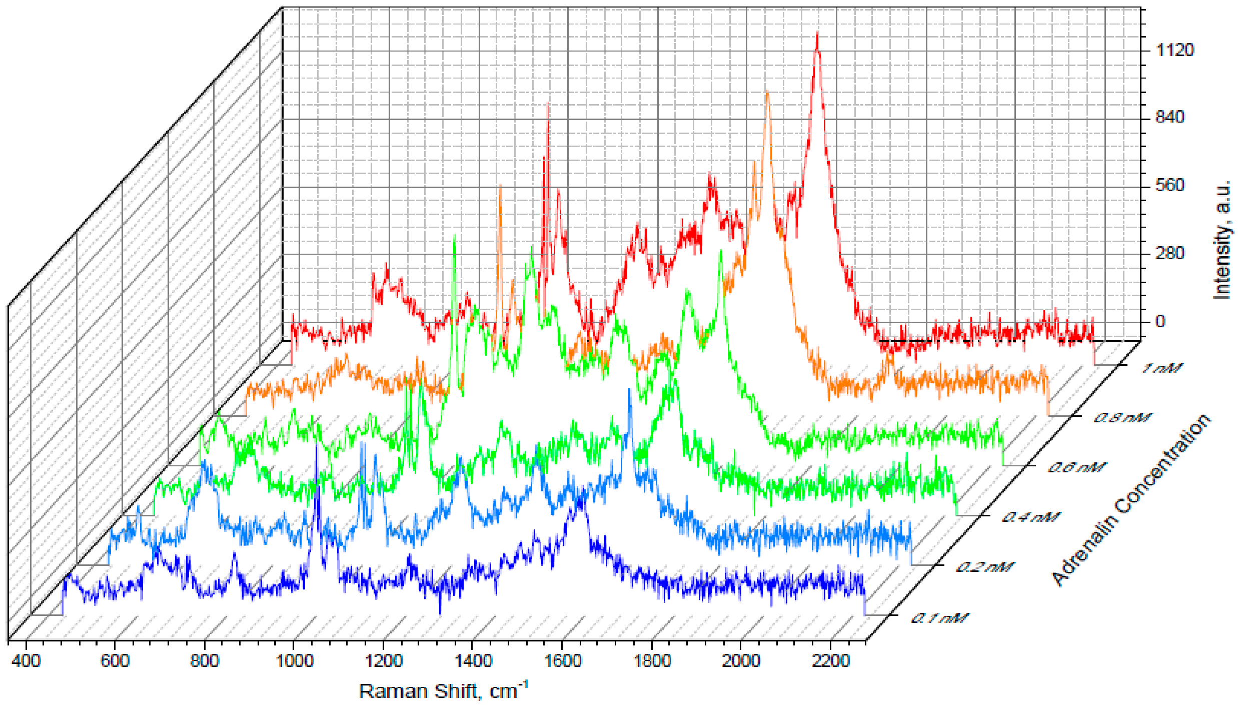

Figure S13. SERS spectra of various AD concentrations applied onto the SERS substrate modified with Cu(II) ions and L^3 (1 mM). All SERS spectra were measured on the silver nanostructured surface coated with the chitosan layer at the resonant 633 nm laser wavelength.

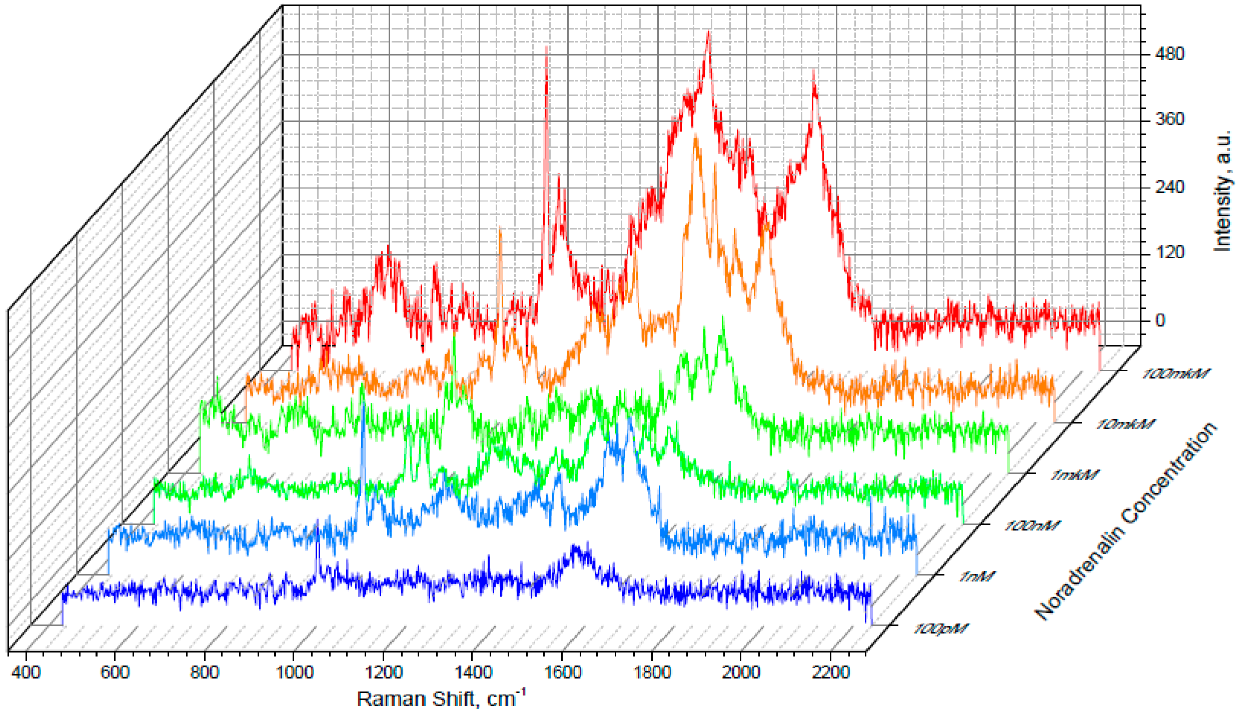

Figure S14. SERS spectra of various NA concentrations applied onto the SERS substrate modified with Cu(II) ions and L^3 (1 mM). All SERS spectra were measured on the silver nanostructured surface coated with the chitosan layer at the resonant 633 nm laser wavelength.

IV. Theoretical Raman Spectra of Catecholamines

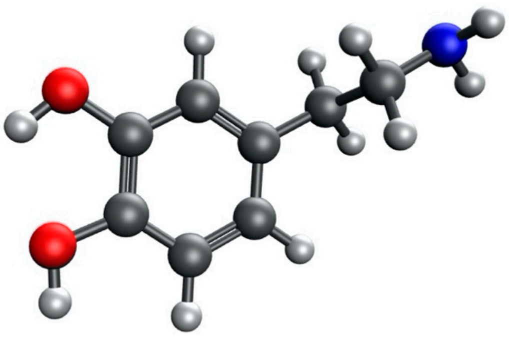

Figure S15. Optimized geometry for dopamine (DA) in “Q-Chem” with PBE0 method and 6-31G* basis.

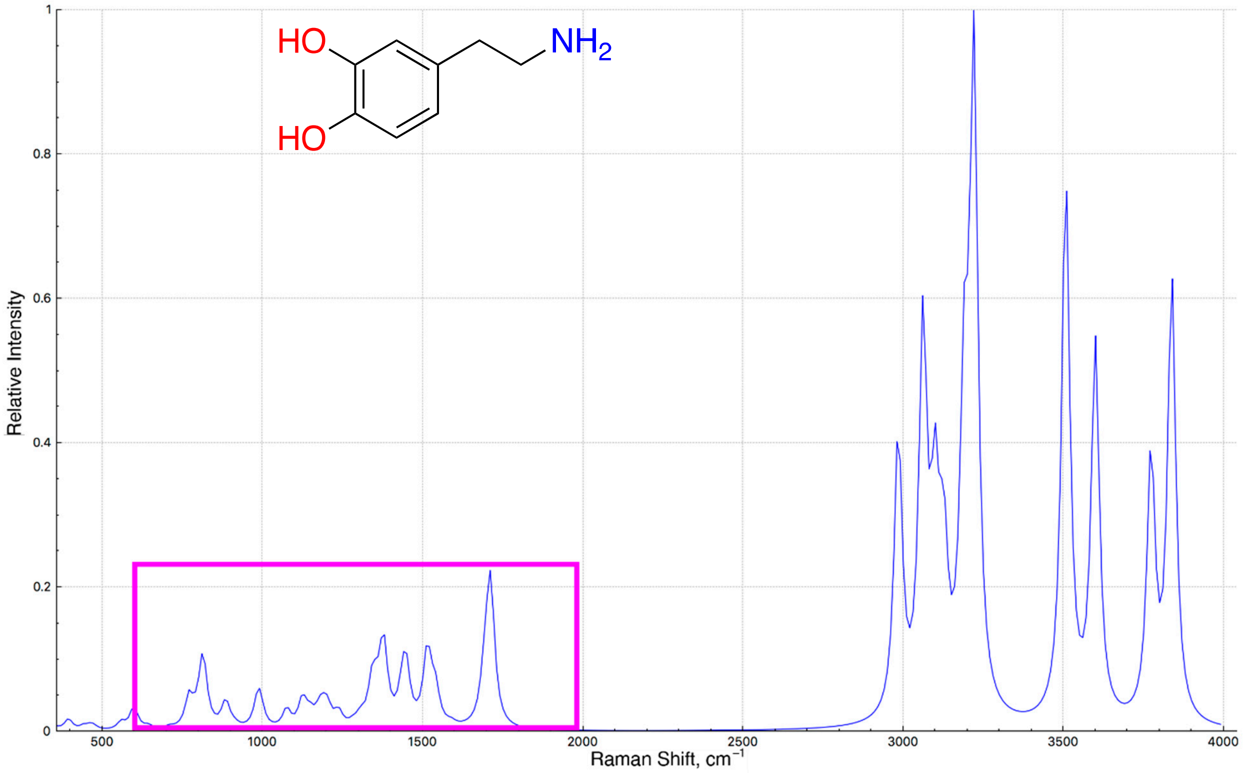

Figure S16. Calculated normalized Raman spectrum for dopamine (DA) in “Q-Chem” with PBE0 method and 6-31G* basis (Lorentzian).

Table S5. Calculated normalized Raman shifts with intensities for dopamine (DA) in “Q-Chem” with PBE0 method and 6-31G* basis (Lorentzian).

Raman Shift, cm^{-1}	Intensity, $Å^4$/amu
558	2.115
**598**	**6.514**
643	1.007
708	0.661
**770**	**8.434**
795	1.373

Raman Shift, cm^{-1}	Intensity, Å^4/amu
**810**	**5.453**
**814**	**14.712**
882	4.926
888	2.699
912	0.818
**984**	**6.222**
**992**	**5.747**
**1075**	**5.129**
1124	7.781
1147	3.435
1173	2.558
1186	4.718
**1202**	**5.205**
1236	4.110
1282	1.047
1306	2.678
**1342**	**10.837**
1355	4.725
**1376**	**14.837**
**1378**	**8.148**
**1434**	**6.004**
**1447**	**16.300**
**1514**	**20.465**
1536	4.371
**1540**	**5.615**
1588	0.954
**1691**	**8.228**
**1701**	**9.219**
**1712**	**33.994**

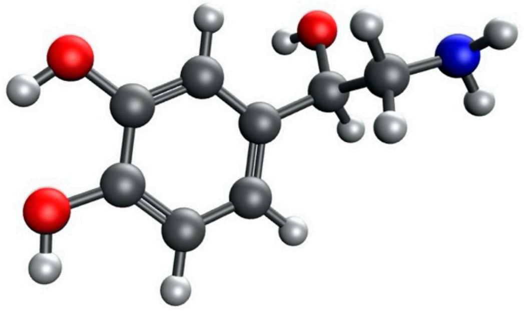

Figure S17. Optimized geometry for noradrenaline (NA) in “Q-Chem” with PBE0 method and 6-31G* basis.

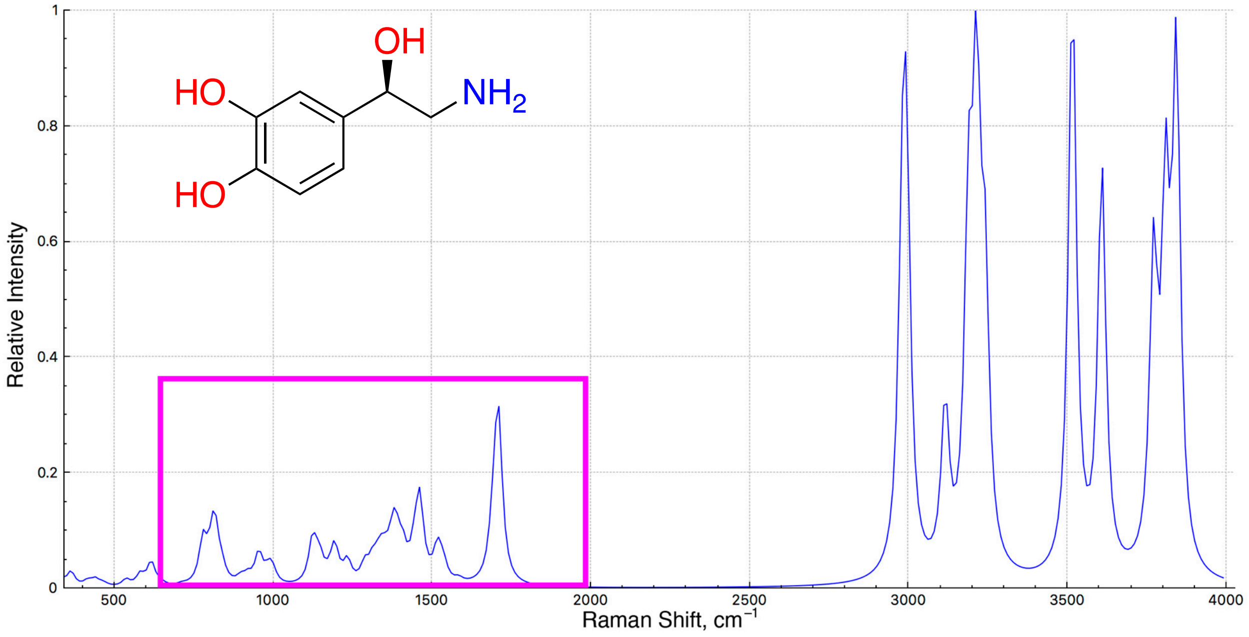

Figure S18. Calculated normalized Raman spectrum for noradrenaline (NA) in "Q-Chem" with PBE0 method and 6-31G* basis (Lorentzian).

Table S6. Calculated normalized Raman shifts with intensities for noradrenaline (NA) in "Q-Chem" with PBE0 method and 6-31G* basis (Lorentzian).

Raman Shift, cm^{-1}	Intensity, Å^4/amu
537	1.709
582	2.856
**616**	**5.961**
645	0.698
713	0.392
**779**	**10.716**
803	3.273
**815**	**14.663**
837	3.458
897	1.632
919	2.034
**954**	**7.749**
986	3.508
998	2.892
**1124**	**10.691**
1140	2.356
1147	4.018
1174	0.806
**1191**	**5.613**
1196	3.852
**1233**	**5.358**
1288	4.317
**1314**	**5.008**
**1336**	**6.367**
1374	4.695

Raman Shift, cm^{-1}	Intensity, $Å^4$/amu
**1386**	**7.202**
**1407**	**9.414**
**1444**	**7.063**
**1461**	**6.317**
**1517**	**19.573**
**1535**	**8.437**
1583	1.063
**1691**	**7.583**
**1702**	**9.313**
**1709**	**34.833**

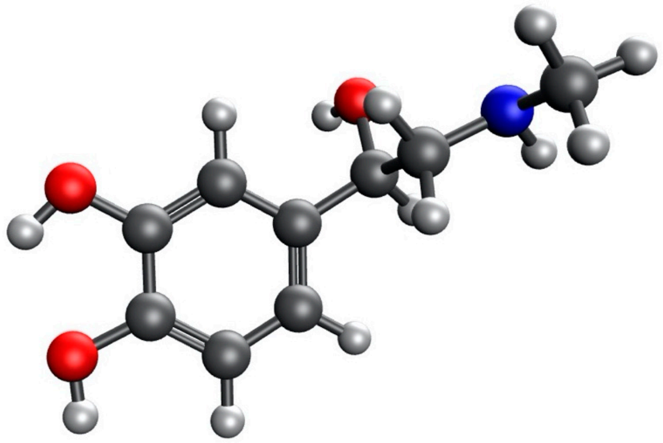

Figure S19. Optimized geometry for adrenaline (AD) in "Q-Chem" with PBE0 method and 6-31G* basis.

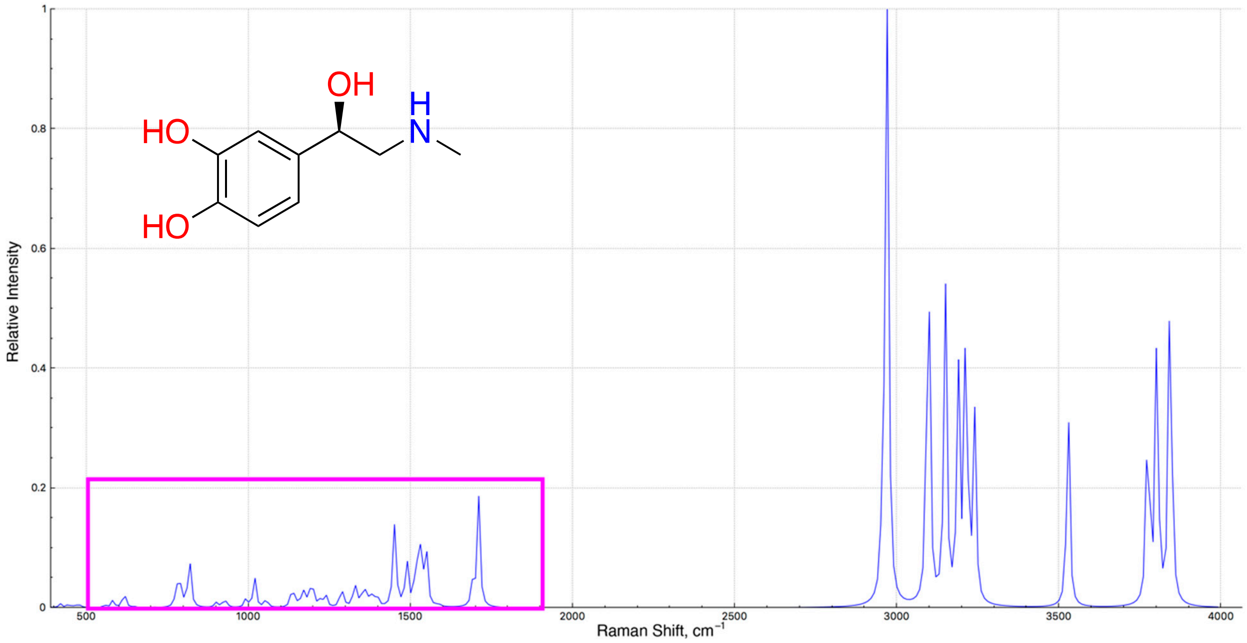

Figure S20. Calculated normalized Raman spectrum for adrenaline (AD) in "Q-Chem" with PBE0 method and 6-31G* basis (Lorentzian).

Table S7. Calculated normalized Raman shifts with intensities for adrenaline (AD) in "Q-Chem" with PBE0 method and 6-31G* basis (Lorentzian).

Raman Shift, cm^{-1}	Intensity, Å^4/amu
556	0.995
582	2.513
**616**	**6.061**
647	0.371
712	0.168
**777**	**5.711**
**787**	**9.441**
807	3.128
**819**	**14.801**
899	1.691
917	1.577
931	2.002
993	3.243
**1018**	**10.641**
1054	3.381
1131	3.572
1142	4.409
**1167**	**6.081**
1176	2.353
**1191**	**5.033**
1199	4.366
1218	2.268
**1237**	**5.491**
1284	3.488
1291	3.873
**1327**	**9.251**
1347	4.332
**1362**	**5.465**
1377	4.399
**1395**	**5.199**
1444	3.299
**1451**	**26.871**
**1488**	**17.017**
1512	1.404
**1517**	**15.238**
**1530**	**14.653**
**1535**	**5.820**
**1548**	**19.546**
1585	0.943
**1690**	**7.091**
**1709**	**37.207**

References

1. Barreto, W.J.; Barreto, S.R.; Ando, R.A.; Santos, P.S.; DiMauro, E.; Jorge, T. Raman, IR, UV-vis and EPR characterization of two copper dioxolene complexes derived from L-dopa and dopamine. *Spectrochim. Acta, Part A* **2008**, *71*, 1419-1424, doi:10.1016/j.saa.2008.04.014.
